# A population-based family clustering study of tic-related obsessive-compulsive disorder

**DOI:** 10.1038/s41380-019-0532-z

**Published:** 2019-10-15

**Authors:** Gustaf Brander, Ralf Kuja-Halkola, Mina A. Rosenqvist, Christian Rück, Eva Serlachius, Lorena Fernández de la Cruz, Paul Lichtenstein, James J. Crowley, Henrik Larsson, David Mataix-Cols

**Affiliations:** 1 Centre for Psychiatry Research, Department of Clinical Neuroscience, Karolinska Institutet, Stockholm, Sweden; 2 Stockholm Health Care Services, Region Stockholm, Stockholm, Sweden; 3 Department of Medical Epidemiology and Biostatistics, Karolinska Institutet, Stockholm, Sweden; 4 Department of Genetics, University of North Carolina, Chapel Hill, NC USA; 5 School of Medical Sciences, Örebro University, Örebro, Sweden

**Keywords:** Psychiatric disorders, Psychology

## Abstract

In the latest edition of the *Diagnostic and Statistical Manual of Mental Disorders (DSM-5)*, obsessive-compulsive disorder (OCD) included a new “tic-related” specifier. However, strong evidence supporting tic-related OCD as a distinct subtype of OCD is lacking. This study investigated whether, at the population level, tic-related OCD has a stronger familial load than non-tic-related OCD. From a cohort of individuals born in Sweden between 1967 and 2007 (*n* = 4,085,367; 1257 with tic-related OCD and 20,975 with non-tic-related OCD), we identified all twins, full siblings, maternal and paternal half siblings, and cousins. Sex- and birth year-adjusted hazard ratios (aHR) were calculated to estimate the risk of OCD in relatives of individuals with OCD with and without comorbid tics, compared with relatives of unaffected individuals. We found that OCD is a familial disorder, regardless of comorbid tic disorder status. However, the risk of OCD in relatives of individuals with tic-related OCD was considerably greater than the risk of OCD in relatives of individuals with non-tic-related OCD (e.g., risk for full siblings: aHR = 10.63 [95% CI, 7.92–14.27] and aHR = 4.52 [95% CI, 4.06–5.02], respectively; *p* value for the difference < 0.0001). These differences remained when the groups were matched by age at first OCD diagnosis and after various sensitivity analyses. The observed familial patterns of OCD in relation to tics were not seen in relation to other neuropsychiatric comorbidities. Tic-related OCD is a particularly familial subtype of OCD. The results have important implications for ongoing gene-searching efforts.

## Introduction

Obsessive-compulsive disorder (OCD) is a heterogeneous disorder [1–3]. This heterogeneity might hinder progress on the identification of etiological factors and the refinement of treatments for the disorder. Attempts to identify homogenous subtypes of the disorder have included, amongst others, early onset OCD [4, 5], symptom dimensions [1, 6], broader underlying constructs such as incompleteness and harm avoidance [7], and comorbidity-based subtypes, such as autism-related OCD [8] or tic-related OCD [9, 10]. While each of these efforts has their own merits, evidence supporting the validity of these subtypes is relatively weak, mainly originating from twin and neuroimaging studies [2, 11–14]. With the exception of hoarding, which is now classified as a separate disorder in the *Diagnostic and Statistical Manual of Mental Disorders (DSM-5)* [15–17], most current research continues to view OCD as a single, homogenous disorder.

Arguably, tic-related OCD is the potential subtype that has received the strongest empirical support [10, 18, 19], which was eventually reflected in the inclusion of a new tic-related specifier in the DSM-5 [15]. Patients endorse the specifier if they have a current or past history of a tic disorder, not necessarily concurrent with their OCD. These patients present with typical clinical characteristics: earlier age of OCD onset [9, 20, 21], greater proportion of males [5, 9, 22–24], higher rates of symmetry and sexual/aggressive obsessions [9, 24, 25], and sensory phenomena preceding the compulsions [22, 26]. Albeit less consistently, studies have also reported a greater number of psychiatric comorbidities [27] and higher rates of attention-deficit/hyperactivity disorder (ADHD) and autism spectrum disorders (ASD) [25] in this patient group. Whether the tic-related OCD subtype is helpful to guide treatment choices is still unclear, with some studies [28, 29], but not all [30, 31], reporting that these patients may respond less well to selective serotonin reuptake inhibitors. Studies are more consistent in showing that patients with tic-related OCD respond equally well to exposure therapy than those with non-tic-related OCD [28, 32].

A few family studies have reported that tic-related OCD may be more familial than non-tic-related OCD [5, 33, 34]. However, these studies were generally small and conducted in specialist OCD and Tourette syndrome clinics which, by definition, may receive more complex comorbid cases. In addition, early age of OCD onset has not sufficiently been taken into account in previous studies. Since early onset OCD has been associated with increased familiality compared with late onset OCD [4, 5], and tic-related OCD has, in turn, been associated with early onset [20, 21], it is important to take this variable into account. It is also possible that the higher familiality of tic-related OCD simply reflects the fact that tic disorders themselves are highly heritable [35, 36]. If this were correct, the co-occurrence of OCD with any other highly heritable neuropsychiatric disorder, such as ADHD or ASD, would result in similarly familial subtypes.

This study leveraged the Swedish national registers to investigate whether tic-related OCD is a particularly familial form of the disorder at the population level. We identified all individuals diagnosed with OCD and classified them into groups with and without tics. We then estimated the risk of OCD across various types of biological relatives in these two groups. In order to confirm the robustness of our results, we accounted for possible differences in age at first OCD diagnosis and examined whether ADHD- and ASD-related OCD were more familial than OCD alone.

## Methods

The study was approved by the Regional Ethical Review Board in Stockholm (reference number 2013/862-31/5). The requirement for informed consent was waived because the study was register-based and the included individuals were not identifiable at any time.

### Data sources

Data were obtained by linking information from Swedish national registers through the unique personal identification numbers assigned to all Swedish citizens at birth or immigration [37]. The registers included were: (1) the Total Population Register, which contains demographic information on all Swedish inhabitants since 1968; (2) the National Patient Register, which covers inpatient hospital admissions since 1969 and outpatient specialist care since 2001, with diagnoses based on the *International Classification of Diseases (ICD)*, eighth (ICD-8; 1969–1986), ninth (ICD-9; 1987–1996), and tenth (ICD-10; 1997–2013) revisions [38]; (3) the Prescribed Drug Register, which contains records of all dispensed medications in Sweden since July 1, 2005; (4) the Multi-Generation Register, which contains information on 100% of mothers and 98% of fathers of individuals born after 1961; [39] (5) the Migration Register, containing information about emigration and immigration in Sweden [40]; and (6) the Cause of Death Register, including dates of all deaths since 1961.

### Study cohort, exposure variables, and outcome variables

We identified all individuals born in Sweden between January 1, 1967 and December 31, 2007 from the Total Population Register. Using the Multi-Generation Register, we then created sub-cohorts consisting of all twins (both monozygotic and dizygotic combined), full siblings, maternal and paternal half siblings, and cousins from the birth cohort. For each sub-cohort, we followed everyone from birth until the first date of a recorded OCD diagnosis, emigration, death, or the end of the follow-up (December 31, 2013), whichever came first. Individuals who died or emigrated before the age of 6 and individuals with conflicting migration dates were excluded from the study population.

Individuals with a lifetime diagnosis of OCD registered between January 1, 1967, and December 31, 2013 were identified from the National Patient Register. We then identified those with a lifetime diagnosis of a tic disorder in addition to the OCD diagnosis (Supplementary Table 1). A lifetime OCD diagnosis, with or without a tic disorder diagnosis, constituted the exposure, and a recorded OCD diagnosis in the relative, independent of a tic diagnosis, constituted the outcome. The Swedish OCD and tic disorder codes have been previously validated [41]. While the tic disorder codes were valid across all editions of ICD, the OCD codes were particularly valid in ICD-10.

Lifetime comorbid psychiatric disorders were also extracted from the National Patient Register and defined as at least one registered diagnosis during the follow-up time. Comorbidities included: (1) ADHD, (2) ASD, (3) schizophrenia and psychotic disorders, (4) bipolar disorder, (5) mood disorders, (6) anxiety disorders, (7) intellectual disability, (8) epilepsy, and (9) organic brain disorder. To avoid false negatives, additional ADHD cases were identified from the Prescribed Drug Register by selecting individuals who had been prescribed specific ADHD medication (Supplementary Table 1).

### Statistical analyses

#### Descriptive statistics and clinical characteristics

The cohort was divided into individuals with OCD and lifetime comorbid tic disorders (tic-related OCD), individuals with OCD who never received a diagnosis of a tic disorder (non-tic-related OCD), and individuals unaffected by OCD. For those, twins, full siblings, maternal half siblings, paternal half siblings, and cousins were identified and the number of individuals and percentages in each category were calculated. Frequencies and percentages for sex and the psychiatric comorbid disorders listed above were calculated for each group; differences between groups were compared using *χ*^2^ tests. Medians and interquartile ranges (IQR) of age at first diagnosis of OCD and age of mothers and fathers at the time of birth were calculated for each group, as well as means and standard deviations of the total number of comorbid disorders for each group. Between-group differences on continuous variables were compared using linear regressions with cluster-robust standard errors and additionally adjusted for sex and birth year.

#### Familial risks of OCD

For each sub-cohort of relatives (i.e., twins, full siblings, maternal half siblings, paternal half siblings, and cousins), we performed Cox proportional hazards regression analyses, with age as the underlying timescale, to estimate hazard ratios (HR) and 95% confidence intervals (CI) of the association between the rate of OCD in relatives of individuals with either tic-related OCD or non-tic-related OCD, compared with the rate of OCD in relatives of individuals without a recorded OCD diagnosis. Birth year and sex of both the index person and the relative were adjusted for in all analyses. Cluster-robust standard errors were calculated to account for the nonindependence of the family clustered data.

Cumulative incidences of OCD were estimated and Kaplan–Meier curves were plotted (under the assumption of no competing risks, estimated as 1-the Kaplan–Meier estimate of survival function) for full siblings of individuals with tic-related and non-tic-related OCD and for unaffected individuals. Cumulative incidences for other groups of relatives (i.e., twins, half-siblings, and cousins) were not calculated due to power issues.

In further analyses, we repeated the main model, excluding all relatives who had any tic disorder diagnosis themselves in order to ensure that the effect of the association was not confounded by the presence of tics in the relatives.

#### Age at first diagnosis analyses

In order to take the early age of tic-related OCD onset into account, we conducted two additional analyses. First, in the complete birth cohort, we matched every tic-related OCD case to five random non-tic-related OCD cases by age at first diagnosis (±1 year), and excluded all nonmatched non-tic-related OCD cases from the sub-cohorts of relatives. We then independently compared the tic-related OCD group and the age-matched non-tic-related OCD subgroup with unaffected individuals, as in the main analysis. Second, we performed a between-groups comparison of the complete tic-related OCD and non-tic-related OCD groups, stratified by age at first diagnosis.

#### ADHD- and ASD-related OCD groups

We created analogous groups of ADHD-related OCD (i.e., OCD with comorbid ADHD) and ASD-related OCD (i.e., OCD with comorbid ASD) and examined the risks of OCD in their respective relatives. Since both ADHD and ASD are highly comorbid with tic disorders [42, 43], we ran additional models where all relatives with any tic disorder were excluded.

#### Sex differences

Potential sex differences in the associations were investigated by stratifying the main Cox regression model by sex combinations (i.e., male–male, male–female, female–male, and female–female) in full siblings only (due to power issues).

Data were managed and analyzed using SAS statistical software, version 9.4 (SAS Institute Inc). R software version 3.4.1 (R Development Core Team, 2017) was used for graphical representations and estimation of cumulative incidence.

## Results

### Descriptives and clinical characteristics

A total of 4,085,367 individuals born between 1967 and 2007 were included in the study and followed up until December 31, 2013, with a median follow-up time of 25.29 years (IQR, 16.33–36.21). During this time, 22,232 individuals (42.64% males) had a recorded OCD diagnosis. Of these, 5.65% cases (*n* = 1 257; 70.96% males) were tic-related, whereas 94.35% were non-tic-related (*n* = 20 975; 40.94% males) (Table 1).

**Table 1:** Descriptive characteristics of study population separately for individuals with tic-related obsessive-compulsive disorder (OCD), non-tic-related OCD, and individuals unaffected by OCD

	Individuals with tic-related obsessive-compulsive disorder *n* = 1257	Individuals with non-tic-related obsessive-compulsive disorder *n* = 20,975	Individuals unaffected by obsessive-compulsive disorder *n* = 4,063,135
Males, *n* (%)	892 (70.96)	8588 (40.94)	2,089,286 (51.42)
Age at first OCD diagnosis, median (IQR)	15.70 (12.17–21.46)	23.13 (17.81–30.03)	–
Age of mother at birth of offspring, median (IQR)	28.75 (25.08–32.33)	28.25 (24.58–32.25)	28.17 (24.59–32.00)
Age of father at birth of offspring, median (IQR)	31.08 (27.58–35.50)	30.75 (27.00–35.25)	30.66 (27.00–34.84)
Psychiatric comorbidities, mean (SD)	1.94 (1.32)	1.56 (1.24)	0.16 (0.53)
ASD, *n* (%)	459 (36.52)	2777 (13.24)	39,875 (0.98)
ADHD, *n* (%)	686 (54.57)	3558 (16.96)	105,968 (2.61)
Schizophrenia, *n* (%)	106 (8.43)	1673 (7.98)	20,184 (0.50)
Bipolar disorder, *n* (%)	80 (6.36)	1731 (8.25)	23,250 (0.57)
Depression, *n* (%)	394 (31.34)	9295 (44.31)	162,402 (4.00)
Anxiety disorders, *n* (%)	534 (42.48)	11,938 (56.92)	210,226 (5.17)
Intellectual disability, *n* (%)	104 (8.27)	807 (3.85)	30,000 (0.74)
Epilepsy, *n* (%)	53 (4.22)	593 (2.83)	48,890 (1.20)
Organic brain disorder, *n* (%)	19 (1.51)	289 (1.38)	6935 (0.17)
Sub-cohorts of relatives			
Twins, *n* (%)	32 (2.55)	375 (1.79)	74,717 (1.84)
Full siblings, *n* (%)	913 (72.63)	15,608 (74.41)	3,056,839 (75.23)
Maternal half siblings, *n* (%)	257 (20.45)	3783 (18.04)	560,953 (13.81)
Paternal half siblings, *n* (%)	273 (21.72)	3897 (18.58)	608,861 (14.99)
Cousins, *n* (%)	874 (69.53)	14,455 (68.92)	2,693,527 (66.29)

*ADHD* attention-deficit/hyperactivity disorder, *ASD* autism spectrum disorders, *IQR* interquartile range, *OCD* obsessive-compulsive disorder, *SD* standard deviation

Patients with tic-related OCD had a significantly lower median age at first OCD diagnosis (15.70 years), compared with non-tic-related OCD (23.12 years; *p* < 0.0001), even after adjusting for sex and birth year (*p* < 0.0001). The mean number of psychiatric comorbid disorders was significantly higher in the tic-related OCD group, compared with the non-tic-related OCD group (1.94 vs. 1.56 comorbidities, respectively; *p* < 0.0001). Of these, ADHD and ASD were significantly more common in the tic-related OCD group (*p* < 0.0001 for both), as were intellectual disability and epilepsy (*p* < 0.0001 and *p* < 0.01, respectively). In contrast, comorbid mood disorders, anxiety disorders, and bipolar disorder were significantly more common in the non-tic-related OCD group (*p* < 0.0001, *p* < 0.0001, and *p* < 0.05, respectively) (Table 1).

### Familial risks of OCD

The estimated risk of OCD in relatives of individuals with OCD increased proportionally to the degree of genetic relatedness in both groups, indicating that OCD is a familial disorder, regardless of tic disorder status (Table 2). However, the HR were higher across most types of relatives of individuals with tic-related OCD. For example, the HR of OCD in full siblings of tic-related OCD individuals was 10.63 (95% CI, 7.92–14.27), compared with 4.52 (95% CI, 4.06–5.02) in full siblings of individuals with non-tic-related OCD. The risk of OCD was approximately twofold for twins, full siblings, and maternal half siblings of individuals with tic-related OCD, compared with the relatives of non-tic-related OCD (Table 2, rightmost column).

**Table 2:** Estimated risk of obsessive-compulsive disorder (OCD) in relatives of individuals with OCD, stratified by tic disorder status

Relative sub-cohort	Frequencies (%)			Hazard ratios (95% CI)^a^		
	Tic-related OCD *n* = 1257	Non-tic-related OCD *n* = 20,975	Unaffected population *n* = 4,063,135	Risk of OCD in relatives of tic-related OCD, compared with OCD unaffected relatives	Risk of OCD in relatives of non-tic-related OCD, compared with OCD unaffected relatives	Risk of OCD in relatives of tic-related OCD, compared with relatives of non-tic-related OCD
Twins	32 (2.55)	375 (1.79)	74,717 (1.84)	**25.19 (9.20–68.98)**	**13.93 (8.78–22.09)**	2.52 (0.91–7.02)
Full siblings	913 (72.63)	15,608 (74.41)	3,056,839 (75.23)	**10.63 (7.92–14.27)**	**4.52 (4.06–5.02)**	**2.11 (1.55–2.87)**
Maternal half siblings	257 (20.45)	3783 (18.04)	560,953 (13.81)	**3.72 (1.92–7.20)**	**1.87 (1.39–2.52)**	**2.15 (1.12–4.11)**
Paternal half siblings	273 (21.72)	3897 (18.58)	608,861 (14.99)	0.30 (0.04–2.12)	**1.80 (1.31–2.49)**	0.20 (0.03–1.41)
Cousins	874 (69.53)	14,455 (68.92)	2,693,527 (66.29)	**1.58 (1.17–2.13)**	**1.43 (1.29–1.59)**	1.05 (0.77–1.42)

*CI* confidence interval, *OCD* obsessive-compulsive disorder

^a^Model adjusted for sex and birth year of both index person and relative

Bold values indicate statistical significance of HRs

**Fig. 1:**
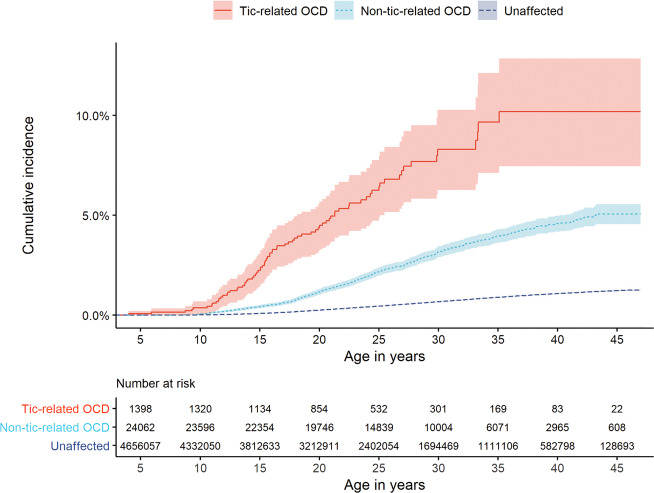
Cumulative incidence under the assumption of no competing risks estimated as the Kaplan–Meier estimate of survival function (with 95% CIs) for OCD in full siblings of individuals with tic-related OCD, with non-tic-related OCD, and unaffected population

In absolute terms, the estimated cumulative incidence of OCD at the end of the follow-up (the oldest individuals being 47 years old) in full siblings of individuals with tic-related OCD was 10.20% (95% CI, 7.46–12.86), compared with 5.06% (95% CI, 4.56–5.56) in full siblings of individuals with non-tic-related OCD (Fig. 1).

When excluding relatives with any tic disorder, the pattern of risk of OCD in relatives of individuals with or without co-occurring tic disorders was similar (Supplementary Table 2) to the main model (Table 2). Similarly, the results remained largely unchanged when the analyses were restricted to individuals with OCD diagnosed with the more valid [41] ICD-10 codes (Supplementary Table 3).

### Age at first diagnosis analyses

After matching the tic-related and non-tic related OCD groups by age at first diagnosis, the median age at first diagnosis in the non-tic-related OCD group was reduced to 16.65 years old (IQR, 13.10–21.71), compared with 15.70 years old (IQR, 12.17–21.46) in the tic-related OCD group (Supplementary Fig. 1). The overall pattern of results remained unchanged, with substantially higher risks in the tic-related OCD group (Table 3).

**Table 3:** Estimated risk of obsessive-compulsive disorder (OCD) in relatives of individuals with OCD, stratified by tic disorder status matched (1:5) by age at first diagnosis of OCD

Relative sub-cohort	Frequencies (%)			Hazard ratios (95% CI)^a^		
	Tic-related OCD *n* = 1257	Non-tic-related OCD *n* = 6,275	Unaffected population *n* = 4,063,135	Risk of OCD in relatives of tic-related OCD, compared with OCD unaffected relatives	Risk of OCD in relatives of non-tic-related OCD, compared with OCD unaffected relatives	Risk of OCD in relatives of tic-related OCD, compared with relatives of non-tic-related OCD
Twins	32 (2.55)	145 (0.69)	74,717 (1.84)	**24.83 (9.05–68.12)**	**11.81 (5.58–25.01)**	2.15 (0.86–5.35)
Full siblings	913 (72.63)	4748 (22.64)	3,056,839 (75.23)	**10.64 (7.93–14.28)**	**5.06 (4.31–5.95)**	**2.02 (1.44–2.83)**
Maternal half siblings	257 (20.45)	1125 (5.36)	560,953 (13.81)	**3.72 (1.92–7.19)**	**2.34 (1.53–3.56)**	1.53 (0.77–3.03)
Paternal half siblings	273 (21.72)	1172 (5.59)	608,861 (14.99)	0.30 (0.04–2.12)	**1.48 (0.89–2.46)**	0.20 (0.03–1.45)
Cousins	874 (69.53)	4450 (21.22)	2,693,527 (66.29)	**1.58 (1.17–2.13)**	**1.46 (1.24–1.70)**	1.08 (0.78–1.48)

*CI* confidence interval, *OCD* obsessive-compulsive disorder

^a^Model adjusted for sex and birth year of both index person and relative

Bold values indicate statistical significance of HRs

Consistently, when we performed a comparison between the tic-related OCD and non-tic-related OCD groups, stratified by age at first OCD diagnosis, the risk of OCD in twins, full siblings, and maternal half siblings, remained almost twofold higher in the tic-related OCD group (Supplementary Table 4).

### ADHD- and ASD-related OCD groups

Full siblings of individuals with ADHD-related OCD were at about 40% increased risk of having OCD compared with full siblings of individuals with non-ADHD-related OCD. This difference, however, was attenuated and no longer statistically significant when excluding all relatives with tic disorders (Supplementary Table 5). No other increases in risk were observed in the ADHD- and ASD-related OCD groups compared with non-ADHD- and non-ASD-related OCD, respectively (Supplementary Tables 5 and 6).

As the diagnoses for ADHD and ASD were introduced in ICD-9, and in order to control for exposure misclassification, we conducted sensitivity analyses running the models for ADHD- and ASD-related OCD on a restricted birth cohort starting in 1987 when ICD-9 was introduced in Sweden. For comparison, this model was also run on tic-related OCD. The observed increased risk of OCD in full siblings of individuals with ADHD-related OCD increased to 65% compared with full siblings of non-ADHD-related OCD. In this younger cohort, the risk of OCD in full siblings of individuals with tic-related OCD was 2.2 times higher than in full siblings of individuals with non-tic-related OCD (Supplementary Table 7).

### Sex differences

When stratifying by sex combinations in full siblings, the HR were still higher for tic-related OCD than for non-tic-related across all combinations (Supplementary Table 8).

## Discussion

This register-based study confirmed the clinical observations of many previous specialist clinic-based studies [20] in a population-based cohort. Indeed, compared with individuals with OCD and no history of tics, individuals with tic-related OCD had earlier age at first OCD diagnosis [20, 21], were more likely males [5, 22–24], had a higher number of comorbidities [27], and, in particular, higher rates of ADHD and ASD [25], but also epilepsy and intellectual disability. Conversely, compared with individuals with tic-related OCD, individuals with non-tic-related OCD had higher rates of comorbid anxiety disorders, mood disorders, and bipolar disorder, also in line with previous work [25, 44]. The fact that many of the clinical characteristics predictably differed between the tic-related and non-tic-related patients, in line with previous knowledge, provides confidence in the validity of these phenotypes at the Swedish population level.

The estimated risk of OCD (with or without tics) was significantly greater in relatives of individuals with OCD than in relatives of individuals from the general population [45]. In both groups, the risk increased with closer genetic relatedness, indicating that OCD is a familial and heritable disorder, regardless of comorbid tic disorder status. However, the risk of OCD in relatives of individuals with tic-related OCD was considerably higher than the risk of OCD in relatives of individuals with non-tic-related OCD. For example, full siblings of individuals with tic-related OCD were ten times more likely to have an OCD diagnosis, compared with full siblings of individuals without OCD, whereas full siblings of individuals with non-tic-related OCD were about four times more likely to have OCD. Directly comparing the tic- and non-tic-related OCD groups with one another revealed more than twofold elevated risk of OCD in twins, full siblings, and maternal half siblings of individuals with tic-related OCD. Additional analyses excluding relatives who had tics themselves did not significantly modify the results.

While the Swedish registers do not provide information on age of onset, we used age at first diagnosis as a proxy. When patients in the tic-related and non-tic-related groups were closely matched on age at first OCD diagnosis and compared with unaffected individuals, our results remained virtually unchanged, with tic-related OCD still being more familial than non-tic-related OCD. In an additional analysis comparing the OCD groups (with and without tics) with one another, while stratifying by age at first diagnosis, the risks of OCD in twins, full siblings, and maternal half siblings of tic-related OCD were still almost twice as high as the same relatives of individuals with non-tic-related OCD. Collectively, these results indicate that the presence of comorbid tics per se explains most of the increased familiality we observe in the population.

Another explanation of the greater familiality of tic-related OCD could be that tic disorders are highly heritable themselves [35, 36, 46]. From this perspective, the co-occurrence of OCD with any other highly heritable disorder would result in similarly familial subtypes. To explore this possibility, we applied the same design to individuals with OCD and comorbid ADHD or ASD, disorders that are at least as heritable as tic disorders [47, 48]. An increased risk of OCD emerged for full siblings of individuals with ADHD-related OCD, compared with full siblings of individuals without comorbid ADHD. However, when excluding tics in relatives to avoid confounding, this risk was attenuated and no longer significant. When restricting the birth cohort to 1987–2007, the observed risk for full siblings of individuals with ADHD-related OCD compared with non-ADHD-related OCD increased, but was still small in comparison with the same risk in tic-related OCD in the same birth year-restricted cohort, indicating a cohort effect. There was no elevated risk of OCD in relatives with ASD and comorbid OCD in any of the analyses. Hence, the increased familiality in tic-related OCD does not seem to be exclusively explained by the comorbidity with a highly heritable neuropsychiatric disorder. The results are also compatible with the fact that OCD and chronic tic disorders are highly genetically correlated (*r* = 0.43), whereas neither ADHD nor ASD are currently known to be genetically correlated with OCD [49].

The results may have important implications for future research, including, but not limited to, current gene-searching efforts. First, exclusion of patients with tic disorders from GWAS studies of OCD could potentially result in a cohort of genetically more homogeneous patients for study. Similarly, tic-related OCD may constitute a genetically interesting subgroup for study. For example, given that Tourette syndrome may be associated with higher rates of copy number variants (CNVs) than OCD [50–53], it would be highly interesting to study CNVs in this particular subgroup of patients. Our results further highlight the importance of careful phenotyping of OCD samples included in genetic studies [54]. Tic-related status is one of the phenotypic characteristics that are most promising in this respect and researchers should strive to systematically assess current and past tic disorder status in their OCD cohorts.

### Strengths and limitations

This is the first population-based family study of tic-related OCD. Major strengths included nationwide data on over four million people prospectively followed up for several decades, and sufficient statistical power to estimate risks across several types of relatives. Nationwide inclusion of patients diagnosed across both specialist and regular psychiatric clinics helped minimize selection and recall biases. Swedish ICD codes for OCD and tic disorders are overall highly valid and reliable [41]. Taking age at first OCD diagnosis into account and examination of other possible familial subtypes based on comorbidity with ADHD and ASD are other unique features of this study.

However, the results should be interpreted in the context of some limitations. Despite the nationwide coverage of the Swedish registers, our cohort does not represent the totality of all OCD and tic disorders patients in Sweden. Many individuals with OCD or tics do not seek help, particularly when symptoms are mild. The coverage of the National Patient Register incomplete before 2001, and patients diagnosed by general practitioners and other nonspecialists are not included. Thus, it is possible that our results may not generalize to less severe patient samples. Some of the groups of relatives were too small to yield precise estimates. For example, information on zygosity was missing for many twins and thus we could not provide separate estimates for monozygotic and dizygotic twins. With additional statistical power, it would have been possible to conduct quantitative genetic modeling analyses and thus attempt to establish whether the increased familiality of tic-related OCD is primarily due to genetic or environmental factors. Age at first OCD diagnosis was used as a proxy of age of disorder onset, because the latter was not available, resulting in the outcome in all groups likely being delayed, due to the lag between disorder onset and registered diagnosis. Finally, with the data at hand, we cannot conclude whether the nature of the tic-related OCD subtype is quantitative, characterized by a higher load of genetic and environmental risks, or qualitative, caused by factors that are not present in either OCD or tic disorders separately. Future genetic research may elucidate this further.

## Conclusions

The results of the study support the validity of the DSM-5 tic-related OCD specifier. Carefully selecting more homogeneous groups of individuals, according to tic-related status, may help guide future gene-searching efforts.

## Supplementary information

Supplementary material
