# Supplementary material for: A population-based family clustering study of tic-related obsessive-compulsive disorder
Source: Mol Psychiatry. 2019 Oct 15;26(4):1224–33. doi: 10.1038/s41380-019-0532-z (PMC7985024; doi:10.1038/s41380-019-0532-z)

Supplementary Table 1. ICD codes for psychiatric disorders.

Disorder	ICD-8 codes	ICD-9 codes	ICD-10 codes
**Obsessive-compulsive disorder**	300.3	300D	F42
**Tourette's and tic disorders**	306.2	307C	F950: Transient tic disorder<br>F951: Chronic motor or vocal tic disorder<br>F952: Tourette's syndrome<br>F958: Other tic disorders<br>F959: Unspecified tic disorder
**Attention-Deficit/Hyperactivity Disorder**[a]	-	314W, 314X	F90
**Autism spectrum disorders**	-	299A	F840, F841, F845, F848, F849
**Schizophrenia and psychotic disorders**	291, 295, 296.99, 297-299	291, 292, 295, 296X, 297-299 (except 299A)	F20-F23, F25, F28, F29, F32.2, F10-F19 subsection .5
**Bipolar disorder**	296.0, 296.1, 296.3, 296.8, 296.9	296A, 296C, 296D, 296E	F30, F31
**Mood disorders (except bipolar disorder)**	296.2, 300,4	296B, 300E, 311	F32-F34 (except F32.3), F38, F39
**Anxiety disorders (except OCD)**	300.2	300A, 300C, 308, 309	F40, F41, F43
**Intellectual disability**	310-315	317-319	F7
**Epilepsy**	345	345	G40
**Organic brain disorder**	290, 293, 294	290, 292, 293, 294	F0

ADHD attention deficit/hyperactivity disorder, *OCD* obsessive-compulsive disorder

[a] Individuals with ADHD were also identified by prescription of ADHD medication, specifically Amfetamine (ATC code: N06BA01), Dexamfetamine (N06BA02), Methylphenidate (N06BA04), and Atomoxetine (N06BA09)

Supplementary Table 2. Estimated risk of obsessive-compulsive disorder (OCD) in relatives of individuals with OCD, stratified by comorbid tic disorder status (excluding relatives with a history of tics)

Relative sub-cohort	Frequencies (%)			Hazard ratios (95% CI)[a]		
	Tic-related OCD N=1257	Non-tic-related OCD N=20,975	Unaffected population N=4,063,135	Risk of OCD in relatives of tic-related OCD, compared to OCD unaffected relatives	Risk of OCD in relatives of non-tic-related OCD, compared to OCD unaffected relatives	Risk of OCD in relatives of tic-related OCD, compared to relatives of non-tic-related OCD
**Twins**	29 (2.31)	370 (1.76)	74,510 (1.83)	**29.39 (10.83-79.77)**	**13.24 (8.02-21.86)**	2.86 (0.89-9.19)
**Full siblings**	884 (70.33)	15,546 (74.12)	3,052,514 (75.13)	**7.40 (5.60-9.80)**	**4.39 (3.92-4.91)**	**1.53 (1.12-2.10)**
**Maternal half siblings**	254 (20.21)	3767 (17.96)	559,690 (13.77)	**3.24 (1.63-6.44)**	**1.79 (1.30-2.48)**	1.97 (0.94-4.13)
**Paternal half siblings**	272 (21.64)	3883 (18.51)	607,692 (14.96)	0.32 (0.05-2.28)	**1.92 (1.38-2.66)**	0.20 (0.03-1.53)
**Cousins**	874 (69.53)	14,455 (68.92)	2,693,242 (66.28)	**1.60 (1.18-2.16)**	**1.42 (1.27-1.59)**	1.08 (0.78-1.49)

CI confidence interval, *OCD* obsessive-compulsive disorder

[a] Model adjusted for sex and birth year of both index person and relative

Note: significant HRs are marked in bold

Supplementary Table 3. Estimated risk of obsessive-compulsive disorder (OCD) in relatives of individuals with OCD, stratified by comorbid tic disorder status (restricted to individuals diagnosed with ICD-10 OCD codes)

Relative sub-cohort	Frequencies (%)			Hazard ratios (95% CI)[a]		
	Tic-related OCD<br>N=1247	Non-tic-related OCD<br>N=20,636	Unaffected population<br>N=4,063,484	Risk of OCD in relatives of tic-related OCD, compared to OCD unaffected relatives	Risk of OCD in relatives of non-tic-related OCD, compared to OCD unaffected relatives	Risk of OCD in relatives of tic-related OCD, compared to relatives of non-tic-related OCD
**Twins**	32 (2.57)	372 (1.80)	74,720 (1.84)	**25.37 (9.24-69.67)**	**14.12 (8.90-22.41)**	2.51 (0.90-6.96)
**Full siblings**	904 (72.49)	15,388 (74.57)	3,057,068 (75.23)	**10.84 (8.08-14.55)**	**4.51 (4.05-5.03)**	**2.15 (1.58-2.92)**
**Maternal half siblings**	257 (20.61)	3725 (18.05)	561,011 (13.81)	**3.78 (1.95-7.32)**	**1.94 (1.44-2.62)**	**2.12 (1.11-4.06)**
**Paternal half siblings**	272 (21.81)	3853 (18.67)	608,906 (14.98)	0.30 (0.04-2.15)	**1.76 (1.26-2.46)**	0.20 (0.03-1.44)
**Cousins**	865 (69.37)	14,276 (69.18)	2,693,715 (66.29)	**1.53 (1.12-2.08)**	**1.43 (1.28-1.59)**	1.01 (0.74-1.38)

CI confidence interval, *OCD* obsessive-compulsive disorder

[a] Model adjusted for sex and birth year of both index person and relative

Note: significant HRs are marked in bold

Supplementary Table 4. Estimated risk of obsessive-compulsive disorder (OCD) in relatives of individuals with OCD, stratified by tic disorder status and by age at first diagnosis

Relative sub-cohort	Frequencies (%)		Hazard ratios (95% CI)[a]
	Tic-related OCD N=1257	Non-tic-related OCD N=20,975	Risk of OCD in relatives of tic-related OCD, compared to relatives of non-tic-related OCD
**Twins**	32 (2.55)	375 (1.79)	1.89 (0.66-5.45)
**Full siblings**	913 (72.63)	15,608 (74.41)	**1.94 (1.43-2.63)**
**Maternal half siblings**	257 (20.45)	3783 (18.04)	1.81 (0.88-3.72)
**Paternal half siblings**	273 (21.72)	3897 (18.58)	0.23 (0.03-1.85)
**Cousins**	874 (69.53)	14,455 (68.92)	0.97 (0.71-1.32)

CI confidence interval, *OCD* obsessive-compulsive disorder

[a] Model adjusted for sex and birth year of both index person and relative

Note: significant HRs are marked in bold

Supplementary Table 5. Estimated risk of obsessive-compulsive disorder (OCD) in relatives of individuals with OCD, stratified by comorbid ADHD status

Relative sub-cohort	Frequencies (%)			Hazard ratios (95% CI)[a]		
	ADHD-related OCD N=4244	Non-ADHD-related OCD N=17,988	Unaffected population N=4,063,135	Risk of OCD in relatives of ADHD-related OCD, compared to OCD unaffected relatives	Risk of OCD in relatives of non-ADHD-related OCD, compared to OCD unaffected relatives	Risk of OCD in relatives of ADHD-related OCD, compared to relatives of non-ADHD-related OCD
**Twins**	80 (1.89)	327 (1.82)	74,717 (1.84)	**13.60 (5.36-34.53)**	**14.82 (9.15-23.99)**	1.12 (0.43-2.96)
**Full siblings**	3017 (71.09)	13,504 (75.07)	3,056,839 (75.23)	**6.63 (5.46-8.05)**	**4.43 (3.95-4.96)**	**1.41 (1.15-1.74)**
**Maternal half siblings**	1090 (25.68)	2950 (16.40)	560,953 (13.81)	**2.16 (1.39-3.35)**	**1.91 (1.39-2.63)**	1.15 (0.74-1.78)
**Paternal half siblings**	1088 (25.64)	3082 (17.13)	608,861 (14.99)	1.47 (0.91-2.38)	**1.78 (1.27-2.49)**	0.90 (0.56-1.45)
**Cousins**	2957 (69.67)	12,372 (68.78)	2,693,527 (66.29)	**1.56 (1.30-1.87)**	**1.41 (1.26-1.58)**	1.08 (0.90-1.30)
**Twins**[b]	77 (1.81)	322 (1.79)	74,510 (1.83)	**14.98 (5.90-38.05)**	**13.91 (8.41-23.03)**	1.30 (0.48-3.57)
**Full siblings**[b]	2984 (70.31)	13,446 (74.75)	3,052,514 (75.13)	**5.44 (4.49-6.60)**	**4.34 (3.86-4.88)**	1.20 (0.97-1.48)
**Maternal half siblings**[b]	1084 (25.54)	2937 (16.33)	559,690 (13.77)	**1.80 (1.13-2.87)**	**1.90 (1.36-2.66)**	0.97 (0.59-1.58)
**Paternal half siblings**[b]	1083 (25.52)	3072 (17.08)	607,692 (14.96)	1.59 (0.98-2.57)	**1.88 (1.34-2.64)**	0.92 (0.58-1.48)
**Cousins**[b]	2957 (69.67)	12,372 (68.78)	2,693,242 (66.28)	**1.53 (1.27-1.85)**	**1.41 (1.26-1.58)**	1.06 (0.87-1.29)

ADHD attention deficit/hyperactivity disorder, *CI* confidence interval, *OCD* obsessive-compulsive disorder

[a] Model adjusted for sex and birth year of both index person and relative
[b] All relatives with tics excluded

Note: significant HRs are marked in bold

Supplementary Table 6. Estimated risk of obsessive-compulsive disorder (OCD) in relatives of individuals with OCD, stratified by comorbid autism spectrum disorders (ASD) status

Relative sub-cohort	Frequencies (%)			Hazard ratios (95% CI)[a]		
	ASD-related OCD N=3236	Non-ASD-related OCD N=18,996	Unaffected population N=4,063,135	Risk of OCD in relatives of ASD-related OCD, compared to OCD unaffected relatives	Risk of OCD in relatives of non-ASD-related OCD, compared to OCD unaffected relatives	Risk of OCD in relatives of ASD-related OCD, compared to relatives of non-ASD-related OCD
**Twins**	74 (2.29)	333 (1.75)	74,717 (1.84)	**14.01 (5.55-35.38)**	**14.72 (9.09-23.83)**	1.03 (0.40-2.67)
**Full siblings**	2360 (72.93)	14,161 (74.55)	3,056,839 (75.23)	**5.66 (4.48-7.16)**	**4.67 (4.19-5.21)**	1.12 (0.87-1.43)
**Maternal half siblings**	625 (19.31)	3415 (17.98)	560,953 (13.81)	**2.35 (1.39-3.97)**	**1.91 (1.40-2.59)**	1.24 (0.71-2.19)
**Paternal half siblings**	629 (19.44)	3541 (18.64)	608,861 (14.99)	1.64 (0.85-3.16)	**1.71 (1.23-2.37)**	1.11 (0.60-2.05)
**Cousins**	2199 (67.95)	13,130 (69.12)	2,693,527 (66.29)	**1.40 (1.12-1.76)**	**1.45 (1.30-1.61)**	0.95 (0.76-1.19)
**Twins[2]**	71 (2.19)	328 (1.73)	74,510 (1.83)	**10.97 (4.50-26.74)**	**14.79 (9.00-24.31)**	0.81 (0.31-2.11)
**Full siblings[2]**	2338 (72.25)	14,092 (74.18)	3,052,514 (75.13)	**4.76 (3.75-6.02)**	**4.49 (4.01-5.03)**	0.99 (0.77-1.27)
**Maternal half siblings[2]**	619 (19.13)	3402 (17.91)	559,690 (13.77)	**2.07 (1.23-3.50)**	**1.84 (1.34-2.52)**	1.16 (0.67-2.01)
**Paternal half siblings[2]**	624 (19.28)	3531 (18.59)	607,692 (14.96)	1.78 (0.92-3.41)	**1.81 (1.30-2.52)**	1.14 (0.61-2.14)
**Cousins[2]**	2199 (67.95)	13,130 (69.12)	2,693,242 (66.28)	**1.34 (1.06-1.69)**	**1.45 (1.30-1.62)**	0.90 (0.71-1.14)

ASD autism spectrum disorders, *CI* confidence interval, *OCD* obsessive-compulsive disorder

[a] Model adjusted for sex and birth year of both index person and relative
[b] All relatives with tics excluded

Note: significant HRs are marked in bold

Supplementary Table 7. Estimated risk of obsessive-compulsive disorder (OCD) in relatives of individuals with OCD, stratified by tic disorder, ADHD, and ASD status, in all individuals born between 1987 and 2007

Relative sub-cohort	Frequencies (%)			Hazard ratios (95% CI)[a]		
	ADHD-related OCD N=2426	Non-ADHD-related OCD N=7219	Unaffected population N=2,103 920	Risk of OCD in relatives of ADHD-related OCD, compared to OCD unaffected relatives	Risk of OCD in relatives of non-ADHD-related OCD, compared to OCD unaffected relatives	Risk of OCD in relatives of ADHD-related OCD, compared to relatives of non-ADHD-related OCD
**Twins**	56 (2.31)	182 (2.52)	52,036 (2.47)	**11.96 (3.64-39.33)**	**11.37 (5.55-23.28)**	1.45 (0.45-4.67)
**Full siblings**	1771 (73.00)	5647 (78.22)	1,609,761 (76.51)	**7.99 (6.34-10.08)**	**4.47 (3.78-5.28)**	**1.65 (1.27-2.13)**
**Maternal half siblings**	616 (25.39)	1193 (16.53)	299,504 (14.24)	**2.76 (1.63-4.67)**	**1.79 (1.17-2.72)**	1.53 (0.91-2.59)
**Paternal half siblings**	622 (25.64)	1315 (18.22)	321,479 (15.28)	0.97 (0.44-2.15)	1.32 (0.77-2.25)	0.80 (0.32-1.99)
**Cousins**	1744 (71.89)	5335 (73.90)	1,402,331 (66.65)	**1.44 (1.13-1.85)**	**1.51 (1.30-1.75)**	0.93 (0.72-1.20)
	ASD-related OCD N=1809	Non-ASD-related OCD N=7836	Unaffected population N=2,103,920	Risk of OCD in relatives of ASD-related OCD, compared to OCD unaffected relatives	Risk of OCD in relatives of non-ASD-related OCD, compared to OCD unaffected relatives	Risk of OCD in relatives of ASD-related OCD, compared to relatives of non-ASD-related OCD
**Twins**	42 (2.32)	196 (2.50)	52,036 (2.47)	**7.43 (1.82-30.26)**	**12.32 (6.27-24.22)**	0.68 (0.18-2.49)
**Full siblings**	1351 (74.68)	6067 (77.42)	1,609,761 (76.51)	**7.18 (5.45-9.46)**	**4.84 (4.12-5.69)**	1.35 (0.99-1.84)
**Maternal half siblings**	370 (20.45)	1439 (18.36)	299,504 (14.24)	**2.97 (1.62-5.44)**	**1.87 (1.19-2.95)**	1.52 (0.70-3.34)
**Paternal half siblings**	364 (20.12)	1573 (20.07)	321,479 (15.28)	0.75 (0.24-2.33)	1.33 (0.79-2.23)	0.61 (0.16-2.26)
**Cousins**	1284 (70.98)	5795 (73.95)	1,402,331 (66.65)	1.09 (0.79-1.49)	**1.59 (1.38-1.83)**	0.66 (0.47-0.91)
	Tic-related OCD	Non-tic-related OCD	Unaffected population	Risk of OCD in relatives of tic-related OCD,	Risk of OCD in relatives of non-tic-related OCD,	Risk of OCD in relatives of tic-related OCD,

	N=892	N=8753	N=2,103,920	compared to OCD unaffected relatives	compared to OCD unaffected relatives	compared to relatives of non-tic-related OCD
**Twins**	25 (2.80)	213 (2.43)	52 036 (2.47)	**7.55 (1.00-56.85)**	**11.85 (6.07-23.12)**	0.85 (0.12-5.83)
**Full siblings**	657 (73.65)	6761 (77.24)	1,609,761 (76.51)	**12.14 (8.78-16.79)**	**4.69 (4.02-5.48)**	**2.22 (1.57-3.13)**
**Maternal half siblings**	181 (20.29)	1628 (18.60)	299,504 (14.24)	**4.55 (2.32-8.94)**	**1.85 (1.23-2.79)**	**2.48 (1.27-4.86)**
**Paternal half siblings**	187 (20.96)	1750 (19.99)	321,479 (15.28)	-	1.36 (0.84-2.18)	-
**Cousins**	631 (70.74)	6448 (73.67)	1,402,331 (66.65)	**1.38 (0.93-2.03)**	**1.51 (1.31-1.73)**	0.86 (0.59-1.26)

ADHD attention deficit/hyperactivity disorder, *ASD* autism spectrum disorders, *CI* confidence interval, *OCD* obsessive-compulsive disorder

[a] Model adjusted for sex and birth year of both index person and relative

Note: significant HRs are marked in bold

Supplementary Table 8. Estimated risk of OCD in full siblings of individuals with OCD, stratified by tic disorder status and by sex of both siblings

	Frequencies (%)			Hazard ratios (95% CI)[a]		
**Sex of siblings**	**Tic-related OCD N=1257**	**Non-tic-related OCD N=20,975**	**Unaffected population N=4,063,135**	**Risk of OCD in relatives of tic-related OCD, compared to OCD unaffected relatives**	**Risk of OCD in relatives of non-tic-related OCD, compared to OCD unaffected relatives**	**Risk of OCD in relatives of tic-related OCD, compared to relatives of non-tic-related OCD**
**Male-male**	393 (31.26)	3934 (18.76)	965,920 (23.77)	**13.02 (8.98-18.87)**	**6.32 (5.37-7.45)**	**1.85 (1.18-2.90)**
**Male-female**	400 (31.82)	3836 (18.29)	944,945 (23.26)	**7.56 (5.02-11.39)**	**4.09 (3.46-4.83)**	**1.76 (1.10-2.81)**
**Female-male**	173 (13.76)	5741 (27.37)	930,770 (22.91)	**10.75 (5.78-20.00)**	**4.26 (3.63-5.00)**	**2.39 (1.14-5.04)**
**Female-female**	147 (11.69)	5558 (26.50)	865,580 (21.30)	**13.95 (8.66-22.45)**	**4.15 (3.63-4.75)**	**3.16 (1.84-5.45)**

CI confidence interval, *OCD* obsessive-compulsive disorder

[a] Model adjusted for sex and birth year of both index person and relative

Note: significant HRs are marked in bold

Supplementary Figure 1. Distribution of ages at first registered diagnosis of obsessive-compulsive disorder (OCD) in the tic-related OCD and non-tic-related OCD groups, before (A) and after (B) matching the groups 1:5 by age (+/- 1 year) at first diagnosis of OCD.

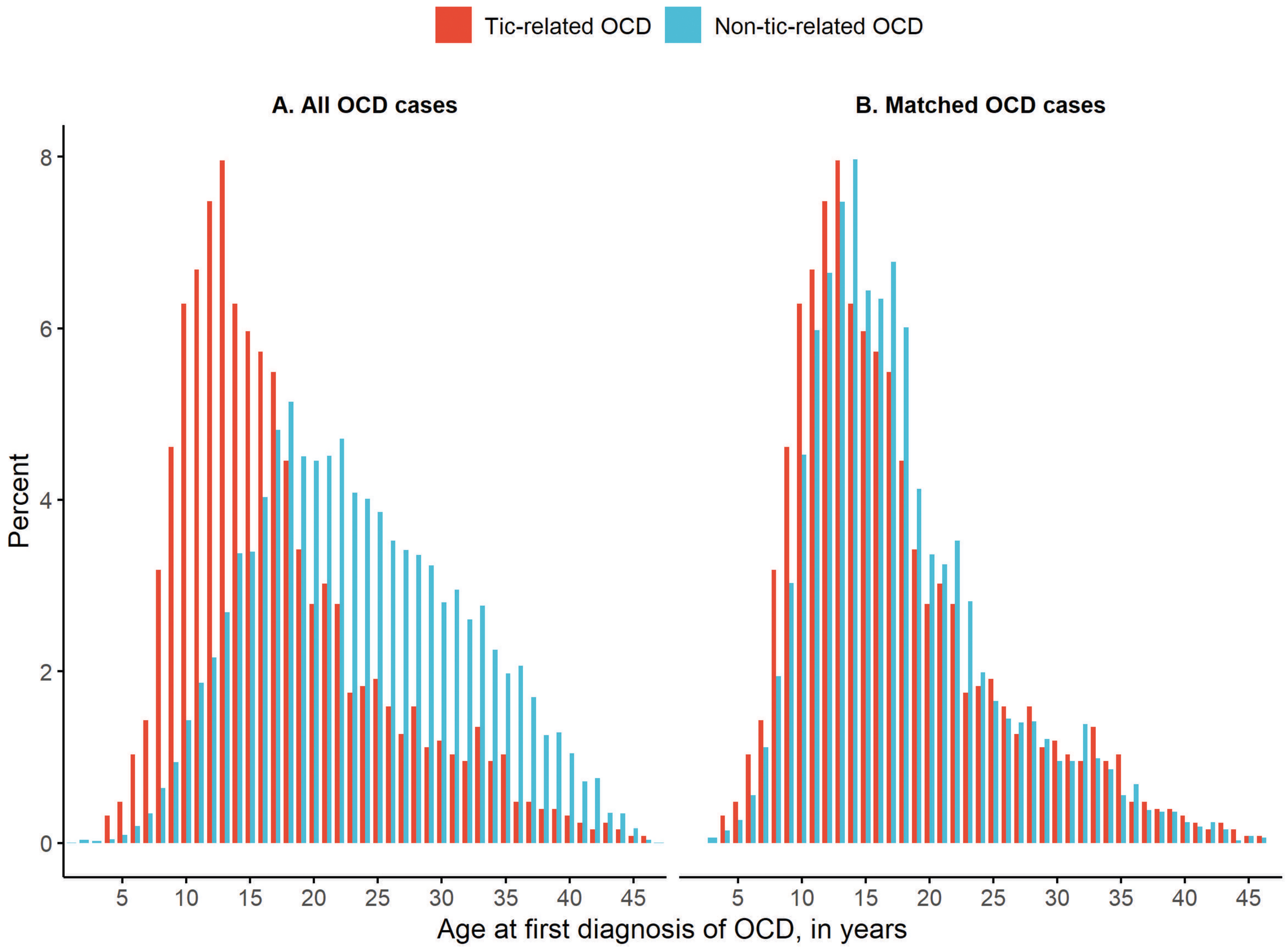

Supplement: Supplementary file 1 — Supplementary material [file 41380_2019_532_MOESM1_ESM.pdf]
